# Analysis of amino acid residues affecting the transcriptional activity of nuclear factor Ya

**DOI:** 10.22099/mbrc.2025.54185.2206

**Published:** 2026

**Authors:** Duhan Tao, Yoshinori Takeuchi, Samia Karkoutly, Zahra Mehrazad Saber, Ye Chen, Tsolmon Mendsaikhan, Rika Saikawa, Yuichi Aita, Yuki Murayama, Akito Shikama, Yukari Masuda, Naoya Yahagi

**Affiliations:** 1 Division of Endocrinology and Metabolism, Department of Medicine, Jichi Medical University, Tochigi 329-0498, Japan; 2 Nutrigenomics Research Group, Institute of Medicine, University of Tsukuba, Ibaraki 305-8575, Japan

**Keywords:** Adipogenesis, Fatty acid synthesis, Transcription

## Abstract

Nuclear factor Y (NF-Y) is a heterotrimeric transcription factor essential for regulating genes involved in lipid metabolism, including fatty acid synthase (Fasn). Although NF-Y activity is known to be dynamically regulated during adipocyte differentiation, the amino acid residues responsible for its transcriptional function remain unclear. In this study, we examined the contribution of nuclear factor Ya (NF-Ya) to DNA-binding activity and transcriptional regulation in 3T3-L1 adipocytes. Using deletion constructs and adenoviral expression systems, we identified a region spanning amino acid residues 184-347 of NF-Ya as critical for acquiring DNA-binding ability during differentiation. Electrophoretic mobility shift assays revealed that NF-Ya undergoes modification within this region, conferring stage-specific DNA-binding activity to the *Fasn* promoter. These findings suggest that post-translational modification of NF-Ya is a key mechanism regulating NF-Y function in adipogenesis. Our work provides novel insights into transcriptional control of lipogenic genes and their relevance to metabolic regulation.

## INTRODUCTION

White adipose tissue (WAT) is the primary organ for energy storage in mammals and plays a central role in whole-body energy homeostasis [1]. In addition to storing excess calories in the form of triglycerides, WAT actively participates in endocrine and metabolic regulation, influencing systemic glucose and lipid metabolism [2]. Dysregulation of WAT function is strongly associated with obesity, insulin resistance, and related metabolic disorders [3].

Upon feeding, fatty acids in adipose tissues are derived from two major sources: circulating triglycerides delivered by lipoproteins and de novo lipogenesis (DNL)[4]. The relative contribution of these two pathways to adipose triglyceride accumulation varies depending on nutritional and hormonal status. While circulating triglycerides provide the bulk of fatty acids, DNL becomes particularly important under conditions of carbohydrate-rich feeding and is closely linked to insulin sensitivity [3]. Indeed, suppression of adipose DNL has been recognized as a hallmark of obesity and insulin resistance.

Fatty acid synthase (FAS; gene name: *Fasn*) is a multifunctional enzyme that synthesizes the 16-carbon saturated fatty acid palmitate from malonyl-CoA and constitutes the rate-limiting step in DNL [5]. *Fasn* expression is tightly regulated at the transcriptional level, and its dysregulation contributes to altered lipid metabolism in obesity and diabetes. Understanding the mechanisms that control *Fasn* transcription in WAT is therefore critical for elucidating the molecular basis of adipose dysfunction.

The transcriptional regulation of *Fasn* in WAT involves multiple transcription factors [2]. We previously reported that among them nuclear factor Y (NF-Y) is of particular importance [6]. NF-Y is a heterotrimeric transcription factor composed of NF-Ya, NF-Yb, and NF-Yc subunits, and it binds to the CCAAT box, a common promoter element in many genes involved in metabolism and cell growth [7, 8]. Emerging evidence suggests that NF-Y activity is influenced by post-translational modifications, which may fine-tune its transcriptional output under different physiological conditions [9]. Given that changes in NF-Y activity could alter *Fasn* transcription, investigating how NF-Y’s DNA-binding ability is regulated at the molecular level is essential. Indeed, modulation of NF-Y - DNA interactions represent a potential mechanism by which adipose tissue adjusts lipogenic gene expression in response to nutritional or hormonal cues.

Structural studies have revealed the three-dimensional architecture of the NF-Y complex bound to DNA, providing insights into its unique histone-fold domains and cooperative binding mechanisms [10]. These findings highlight the critical contribution of specific amino acid residues within the NF-Y subunits to DNA recognition and transcriptional regulation. However, the precise residues that determine NF-Y's DNA binding affinity and transcriptional activity remain incompletely understood.

In this study, we aimed to identify the amino acid residues that are critical for the DNA binding and transcriptional activity of NF-Ya. Using deletion constructs and adenoviral expression systems, we sought to narrow down the structural and functional determinants that govern NF-Y - dependent regulation of *Fasn* and other metabolic genes in WAT. These findings will provide new mechanistic insights into how NF-Y integrates metabolic signals to control lipogenic gene expression.

## MATERIALS AND METHODS

### Cell culture:

3T3-L1 preadipocytes were cultured in high-glucose DMEM supplemented with 10% CS, 8 mg/L D-biotin, and 8 mg/L calcium pantothenate. For undifferentiated 3T3-L1 cells, cultures were maintained below 50% confluence to avoid contact inhibition. For differentiation, cells were grown to 100% confluence (designated day −2), and two days later (day 0), differentiation medium containing 10 µg/mL insulin, 1% 1-methyl-3-isobutylxanthine (IBMX), 1 µM dexamethasone, and 1 µM pioglitazone was applied. On day 2, the medium was replaced with fresh DMEM containing 10 µg/mL insulin and 1 µM pioglitazone. Lipid droplet accumulation became apparent during differentiation, and fully differentiated adipocytes were typically observed by day 6 [6]. At least two repetitions of each experiment were carried out to ensure the elimination of any bias present in each experimental environment.

### Nuclear extraction:

Nuclear extraction was performed as previously described [6]. Cells were resuspended in Buffer I (10 mM HEPES, pH 7.9; 10 mM KCl; 1.5 mM MgCl₂; 1 mM EGTA; 1 mM EDTA; 1 mM DTT; protease inhibitors: PMSF 0.2 mM, leupeptin 1 µg/mL, aprotinin 1 µg/mL, pepstatin A 1 µg/mL). The suspension was passed through a 26-gauge needle 20 times to disrupt the plasma membrane. Nuclei were collected by centrifugation at 6,000 rpm for 5 min at 4˚C. The pellet was extracted in Buffer II (20 mM HEPES, pH 7.9; 25% glycerol; 420 mM NaCl; 1.5 mM MgCl₂; 1 mM DTT; the same protease inhibitors as above) on a rotating platform for 15 min at 4˚C, followed by centrifugation at 15,000 rpm for 20 min at 4˚C. Supernatants (nuclear extracts) were collected, and protein concentrations were determined by the Bradford assay.

### Generation of recombinant adenovirus:

HA-tagged expression plasmids were generated using pcDNA3-HA (Invitrogen) as the backbone. NF-Ya-Full and NF-Ya-184~347(-Short) cDNA fragments were cloned into pcDNA3-HA with the In-Fusion® HD Cloning Kit (TaKaRa). Specifically, the NF-Ya-short fragment was PCR-amplified and inserted into HindIII/XhoI-linearized pcDNA3-HA by In-Fusion, ensuring that the insert was in-frame with the HA start codon and retaining the native NF-Ya stop codon. The primer sequences used for In-Fusion cloning are listed in supplementary Table S1. For adenoviral constructs, NF-Ya-Full and NF-Ya-Short cDNAs were further subcloned into pENTR4-FLAG (Invitrogen) by In-Fusion. The pENTR4-FLAG vector was linearized with BamHI, and inserts were amplified with primers carrying 15 bp overlaps to the digested ends, ensuring in-frame fusion with the N-terminal FLAG tag and retention of the native stop codon. The entry clones were subsequently recombined with pAd CMV/V5-DEST(Invitrogen) using the Gateway® Clonase system to obtain adenoviral plasmids [11, 12].

### Western blotting:

Western blotting was performed as previously described [13]. NF-Ya were detected using anti-NF-Ya (G-2) (SC-17753, mouse monoclonal IgG, Santa Cruz Biotechnology) and anti-mouse IgG (#7076s, Cell Signaling) antibodies. Signals were developed with Western Lightning Plus‑ECL (PerkinElmer) and imaged on an ImageQuant LAS‑4000 (GE Healthcare).

### Electrophoretic Mobility Shift Assay (EMSA):

EMSA was performed as previously described [6]. A double‑stranded inverted CCAAT element (ICE) probe (*Fasn* promotor -110 ~ -86) was prepared by annealing complementary oligonucleotides (5’-GCCCCGACGCTCATT GGC-3’ and 5’-GCCCAGGCCAATGAGCGT-3’) in H‑buffer (TaKaRa). Oligos were heated to 95˚C for 5 min and cooled to RT for 60 min. Probes were labeled with [α‑³²P]dCTP using the Megaprime DNA Labeling System (RPN1606; Amersham Biosciences). Binding reactions (20 µL) containing labeled probe and recombinant proteins (as indicated) were assembled in EMSA binding buffer (10 mM Tris‑HCl, pH 7.5; 100 mM KCl; 0.5 mM EDTA; 1 mM MgCl₂; 5% glycerol; 1 mM DTT; poly[dI‑dC] 30 µg/mL) and incubated on ice for 30 min. Complexes were resolved on 4.6% polyacrylamide gels in 0.5× TBE buffer at 100 V for 60 min. Gels were dried at 85˚C for 2 h, exposed to phosphor screens overnight, and scanned on a BAS‑2500 (Fujifilm).

### In vitro transcription and translation (IVTT):

NF‑Ya, NF‑Yb, and NF‑Yc proteins were generated using the TNT® Quick Coupled Transcription/Translation System (Promega) according to the manufacturer’s instructions. Typical 25 µL reactions contained 20 µL TNT master mix, 0.5 µg plasmid DNA template for NF‑YA, NF‑YB, and NF‑YC [6], and 0.5 µL 1 mM methionine; reactions were incubated at 30 ˚C for 90 min. IVTT products were evaluated by western blotting to confirm expression and to ensure comparable NF‑Y protein levels prior to EMSA.

## RESULTS

**Figure 1:**
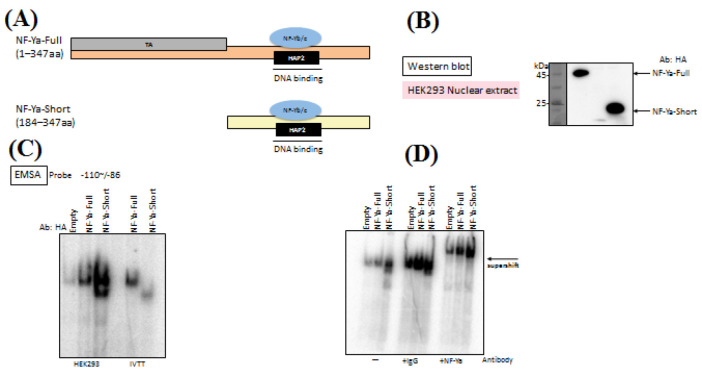
Structural comparison of NF-Ya-Full length and NF-Ya-Short isoforms, and analysis of their expression and DNA binding activity.

To narrow down the possible modified amino acid residues in the NF-Ya protein, we generated a short form of NF-Ya (NF-Ya-Short). Schematic representations of the full-length and short forms were shown in Figure 1A. The HAP2 homology domain can be subdivided into two functional regions, with the N-terminal subdomain mediating protein–protein interactions and the C-terminal subdomain responsible for DNA binding [15].

To assess the relative expression of NF-Ya-Full and NF-Ya-Short, HEK293 cells were transfected with HA-tagged constructs encoding each protein. Nuclear extracts were prepared and analyzed by Western blot using an anti-HA antibody (Fig. 1B). Both NF-Ya-Full and NF-Ya-Short were detected at comparable levels, indicating that deletion of the N-terminal transactivation domain in NF-Ya-Short does not compromise nuclear expression or protein stability.

To compare the DNA-binding activity of NF-Ya-Full and NF-Ya-Short, electrophoretic mobility shift assays (EMSA) were performed using nuclear extracts from transfected HEK293 cells. The probe corresponded to the inverted CCAAT element (ICE), which is located between positions -110 and -86 in the *Fasn* promoter. This element has been identified as critical for mediating the refeeding response in adipocytes [6]. Both NF-Ya-Full and NF-Ya-Short exhibited comparable DNA-binding activity to the ICE probe (Fig. 1C). In vitro translated (IVTT) NF-Ya proteins were included as controls and showed consistent binding patterns, supporting the specificity of the observed protein-DNA interactions.

To confirm that the shifted complex observed in the EMSA corresponds to NF-Ya, a supershift assay was performed (Fig. 1D). Nuclear extracts were incubated with the ICE probe (-110 to -86 of the *Fasn *promoter) in the presence of an anti-NF-Ya antibody. The addition of the antibody resulted in a supershift of the DNA-protein complex, thereby confirming NF-Ya as a component of the binding complex, consistent with previous reports in mature adipocytes [6]. Normal IgG was used as a negative control and did not alter the mobility of the band.

(**A**) Schematic of NF-Ya-Full (upper) and NF-Ya-Short (lower). NF-Ya-Full contains both the N-terminal transcriptional activation (TA) and C-terminal HAP2 domains, whereas NF-Ya-Short lacks the TA domain but retains HAP2 for NF-Yb/c binding. (**B**) Western blot analysis of NF-Ya-Full and NF-Ya-Short in nuclear extracts of transfected HEK293 cells, detected with anti-HA antibody. (**C**) Electrophoretic mobility shift assay (EMSA) of NF-YA-Full and NF-YA-Short in nuclear extracts of HEK293 cells. DNA binding activity to the ICE element of the *Fasn* promoter (probe spanning -110 to -86) was assessed using nuclear extracts from cells transfected with NF-Ya-Full and NF-Ya-Short constructs. In vitro translated (IVTT) NF-Ya proteins were used as controls to validate binding specificity. (**D**) Supershift assay of NF-Ya-Full and NF-Ya-Short in nuclear extracts of HEK293 cells. EMSA was performed with the ICE probe (–110 to –86), and addition of anti-NF-Ya antibody induced a supershift, whereas normal IgG was used as a negative control, thereby identifying the shifted band as NF-Ya.

**Figure 2:**
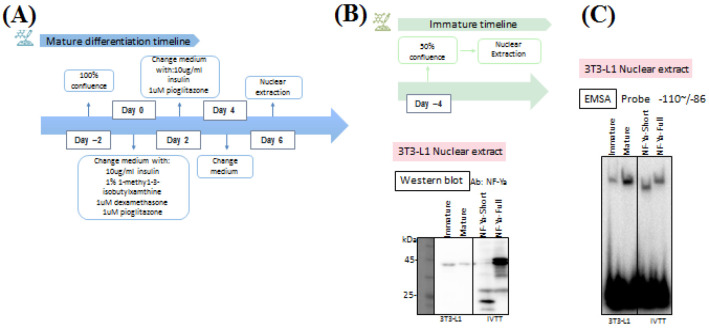
Mature and Immature 3T3-L1 adipocytes.

To investigate NF-Ya regulation during adipocyte differentiation, nuclear extracts were prepared from 3T3-L1 cells at both immature and mature stages. The differentiation protocol is illustrated in Figure 2A. Immature samples were collected at ~50% confluence prior to induction (day -4). For mature adipocytes, differentiation was initiated at day -2, when cells had reached 100% confluence. At day 0, differentiation was induced by changing to medium containing 10 µg/mL insulin, 1% 1-methyl-3-isobutylxanthine (IBMX), 1 µM dexamethasone, and 1 µM pioglitazone. On day 2, the medium was replaced with fresh medium supplemented with 10 µg/mL insulin and 1 µM pioglitazone. On day 4, cells were given a medium change without additional supplements. Nuclear extracts from mature adipocytes were harvested on day 6.

To determine whether NF-Ya expression changes during differentiation, nuclear extracts were analyzed by Western blot using an anti–NF-Ya antibody (Fig. 2B). NF-Ya protein was detected at comparable levels in both immature and mature adipocytes. In vitro translated (IVTT) NF-Ya protein was included as a control to verify immunoblot specificity.

To assess NF-Ya DNA-binding activity, EMSA was performed using the ICE probe (-110 to -86 of the *Fasn* promoter) with nuclear extracts from immature and mature 3T3-L1 cells (Fig. 2C). A strong band of DNA-protein complex was observed in extracts from mature adipocytes, whereas a much weaker band was observed in extracts from immature cells. This finding is consistent with previous reports showing increased protein binding to the -110/-86 probe upon adipocyte maturation [6]. Together with the Western blot data and the IVTT protein control, these results demonstrate that the DNA-binding ability of NF-Ya to the ICE probe significantly increases during the differentiation process of adipocytes.

Western blot analysis showed NF-Ya protein levels, and EMSA revealed differences in DNA binding activity to the ICE element of the *Fasn* promoter. (**A**) Timeline illustrating the differentiation process of 3T3-L1 adipocytes. Mature (differentiated) and Immature (undifferentiated) 3T3-L1 cells were cultured under distinct conditions, and nuclear extracts were prepared at the corresponding time points for subsequent analyses. (**B**) Western blot analysis of NF-Ya expression in Mature and Immature 3T3-L1 adipocytes. Nuclear extracts were prepared from Mature (differentiated) and Immature (undifferentiated) 3T3-L1 cells, and NF-Ya protein levels were detected using anti-NF-Ya antibody. In vitro translated (IVTT) NF-Ya was included as a control. (**C**) Electrophoretic mobility shift assay (EMSA) of NF-Ya in nuclear extracts from Mature (differentiated) and Immature (undifferentiated) 3T3-L1 cells. DNA binding activity to the ICE element of the *Fasn* promoter (probe spanning -110 to -86) was assessed and in vitro translated (IVTT) NF-Ya proteins were used as a control. Despite equal protein levels confirmed by Western blot, EMSA revealed differential DNA binding activity between Mature and Immature cells.

**Figure 3:**
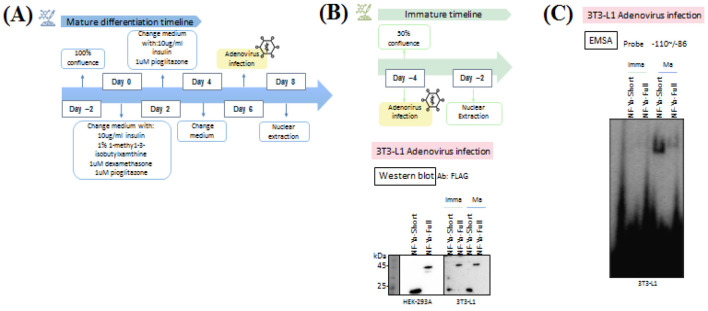
Adenovirus mediated expression of NF-Ya-Full and NF-Ya-Short in 3T3-L1 cells and validation in HEK293 cells.

To investigate the stage-specific DNA-binding ability of NF-Ya-Full and NF-Ya-Short, 3T3-L1 cells were infected with recombinant adenoviruses at both immature and mature stages of differentiation (Fig. 3A). Immature cells were infected at ~50% confluence (day -4) and harvested two days later for nuclear extraction. For mature adipocytes, differentiation was initiated on day -2, when cells had reached 100% confluence. On day 0, differentiation was induced by changing to medium containing 10 µg/mL insulin, 1% 1-methyl-3-isobutylxanthine (IBMX), 1 µM dexamethasone, and 1 µM pioglitazone. On day 2, the medium was replaced with fresh medium supplemented with 10 µg/mL insulin and 1 µM pioglitazone. On day 4, cells received a medium change without additional supplements. Adenoviral infection with NF-Ya-Full or NF-Ya-Short was performed on day 6, and nuclear extracts were collected on day 8.

To confirm the efficiency of the adenoviral constructs, HEK293 cells were first infected and nuclear extracts were analyzed by Western blot using an anti-FLAG antibody. Distinct bands corresponding to NF-Ya-Full and NF-Ya-Short were detected, confirming that the adenoviruses expressed the intended proteins (Fig. 3B). Subsequently, immature and mature 3T3-L1 cells were infected under the conditions described in Figure 3A. Western blot analysis demonstrated robust expression of NF-Ya-Full and NF-Ya-Short in both immature and mature adipocytes, validating efficient adenoviral-mediated expression across differentiation stages.

To determine whether adenovirally expressed NF-Ya-Full and NF-Ya-Short retained DNA-binding activity, EMSA was performed using nuclear extracts from infected 3T3-L1 cells with the ICE probe (-110 to -86 of the *Fasn* promoter). A distinct DNA-protein complex was observed in mature adipocytes infected with either NF-Ya-Full or NF-Ya-Short, whereas no detectable binding was observed in infected immature cells (Fig. 3C). These findings indicate that both adenovirally expressed NF-Ya-Full and NF-Ya-Short have the ability to bind to DNA in mature adipocytes. However, DNA-binding activity is absent in the immature state, despite efficient protein expression.

(**A**) Timeline of adenovirus infection at different stages of 3T3-L1 differentiation (Mature vs. Immature), with nuclear extraction at the indicated time points. (**B**) Western blot analysis of FLAG-tagged NF-Ya-Full and NF-Ya-Short in nuclear extracts from adenovirus infected Mature and Immature 3T3-L1 cells using anti-FLAG antibody, as well as HEK293 cells. (**C**) Electrophoretic mobility shift assay (EMSA) of nuclear extracts from adenovirus infected Mature and Immature 3T3-L1 cells using the ICE probe spanning -110 to -86, showing increased NF-Ya binding during 3T3-L1 differentiation.

## DISCUSSION

In the present study, we clearly demonstrated that NF-Ya acquires DNA-binding ability through modification during the adipocyte differentiation process. Furthermore, we clarified that the modification site is located between amino acid residues 184 and 347. This observation extends our previous reports showing that NF-Y activity is dynamically regulated during adipocyte differentiation and metabolic adaptation [6]. The identification of this domain provides a framework for future biochemical studies aimed at dissecting how NF-Ya structural changes influence its transcriptional activity.

The localization of the modification site between residues 184 and 347 raises the possibility that multiple types of post-translational modifications (PTMs) could be involved. Lysine acetylation, which has been reported to enhance DNA-binding affinity in several transcription factors [16-18], may play a role given the high lysine density within this region. Similarly, serine/threonine phosphorylation is known to regulate DNA-protein interactions of NF-Y complex [19-21]. Future studies using mass spectrometry-based proteomics will be necessary to pinpoint the exact modification and determine its functional consequences in adipocytes.

The upstream regulators responsible for introducing these modifications are also of great interest. Several signaling pathways activated during adipogenesis, including PKA/CREB, MAPK, and PI3K-Akt, have been implicated in the regulation of transcription factor PTMs [22, 23]. Histone acetyltransferases such as p300/CBP, which interact with NF-Y complexes to co-regulate adipogenic genes [24], could also directly acetylate NF-Ya. Identifying these upstream effectors will be critical to understanding how extracellular signals and nutrient status converge on NF-Y to regulate adipocyte gene expression.

Regarding the regulatory mechanism of de novo lipogenesis (DNL) in adipocytes, we have demonstrated that, unlike in the liver, mechanisms independent of sterol regulatory element-binding protein-1 (SREBP-1) are more important in adipocytes [6, 25, 26]. Adipocyte DNL is tightly regulated by the insulin signaling pathway, and insulin resistance — a hallmark of obesity and type 2 diabetes — disrupts glucose and lipid metabolism across multiple tissues. White adipose tissue (WAT), which plays a central role in systemic energy homeostasis, is particularly vulnerable under obese conditions. Accumulating evidence demonstrates that obesity leads to suppression of *Fasn* gene expression in WAT, linking adipose tissue lipogenesis to systemic insulin sensitivity [3, 27-30]. Reduced *Fasn* expression not only reflects altered adipocyte function but also contributes to the metabolic inflexibility observed in insulin-resistant states [2]. Thus, elucidating the molecular mechanisms governing *Fasn* expression in adipose tissue is essential for understanding the pathogenesis of obesity-associated insulin resistance.

In summary, our findings provide novel evidence that NF-Ya undergoes post-translational modification during adipocyte differentiation, conferring DNA-binding activity through a region spanning residues 184-347. This discovery establishes a mechanistic basis for how NF-Ya activity is regulated and highlights potential connections between upstream signaling pathways and transcriptional control in adipogenesis. Further work is needed to identify the exact nature of the modification and its regulatory enzymes, which will not only advance our understanding of NF-Y biology but may also provide therapeutic opportunities for metabolic diseases where NF-Y-dependent transcription is dysregulated.

### Acknowledgements:

This work was supported by MEXT/JSPS KAKENHI Grant Numbers 23116006 (Grant-in-Aid for Scientific Research on Innovative Areas: Crosstalk of transcriptional control and energy pathways by hub metabolites), 23K24760 and 15H03092 (Grant-in-Aid for Scientific Research (B)), and 24K22110 (Grant-in-Aid for Challenging Research (Exploratory)) (to N. Yahagi). This research was also supported by AMED under Grant Number JP23gm1710008 (AMED-CREST) and JP23rea522010 (Healthcare Social Implementation Infrastructure Development Project) (to N. Yahagi).

### Conflict of Interest:

 The authors declare no competing financial and non-financial interests.

### Authors’ Contribution:

N.Y. conceived the experiments. D.T. performed the experiments under the guidance of Y.T. and analyzed the data together with N.Y. D.T. Y.T. and N.Y. co-wrote the paper. All authors discussed the results and commented on the manuscript.
