# Supplementary material for: Analysis of amino acid residues affecting the transcriptional activity of nuclear factor Ya
Source: Mol Biol Res Commun. 2026;15(1):21–9. doi: 10.22099/mbrc.2025.54185.2206 (PMC12673625; doi:10.22099/mbrc.2025.54185.2206)
Supplement: Supplementary file 1 — Table S1 [file mbrc-15-21-s001.pdf]

Molecular Biology Research Communications 2026;15(1):21-29 DOI:10.22099/mbrc.2025.54185.2206 MBRC

Original Article Open Access

Analysis of amino acid residues affecting the transcriptional activity of nuclear factor Ya

Duhan Tao[1,2], Yoshinori Takeuchi[1], Samia Karkoutly[1], Zahra Mehrazad Saber[1], Ye Chen[1,2], Tsolmon Mendsaikhan[1], Rika Saikawa[1], Yuichi Aita[1,2], Yuki Murayama[2], Akito Shikama[2], Yukari Masuda[1], Naoya Yahagi[1,2,*]

1) Division of Endocrinology and Metabolism, Department of Medicine, Jichi Medical University, Tochigi 329-0498, Japan
2) Nutrigenomics Research Group, Institute of Medicine, University of Tsukuba, Ibaraki 305-8575, Japan

Table S1: Infusion primer and sequence for constructing NF-Ya-Full and NF-Ya-Short

**Primer for HA-NF-Ya-Short**	
**NF-Ya-Short F**	5’-CGACTACGCGGGATCCACAGTCCCTGTTTCAGGCAT-3’
**NF-Ya-Short R**	5’- ATTCGGTACCGGATCTTAGGAAACTCGGATGATCT -3’
**Primer for pENTR4-FLAG-NF-Ya-Full**	
**NF-Ya-Full F**	5’-CGATGACAAGGGATCCATGGAGCAGTATACGACAAA-3’
**NF-Ya-Full R**	5’-ATTCGGTACCGGATCTTAGGAAACTCGGATGATCT-3’
**Primer for pENTR4-FLAG-NF-Ya-Short**	
**NF-Ya-Short F**	5’-CGATGACAAGGGATCCACAGTCCCTGTTTCAGGCAT-3’
**NF-Ya-Short R**	5’-ATTCGGTACCGGATCTTAGGAAACTCGGATGATCT-3’

Table S1: Infusion primer and sequence for constructing NF-Ya-Full and NF-YaShort (from 5’→3’).
